# *β*-hydroxy-*β*-methylbutyrate Attenuates Age-Dependent Loss of Flight Ability and Extends Lifespan in Drosophila

**DOI:** 10.3390/ijms26062664

**Published:** 2025-03-15

**Authors:** Ravi Nagori, Jim O. Vigoreaux

**Affiliations:** Department of Biology, University of Vermont, Burlington, VT 05405, USA

**Keywords:** Drosophila, flight muscle, lifespan, HMB, aging, mitochondria

## Abstract

β-hydroxy-β-methylbutyrate (HMB) has been shown to enhance muscle function and strength in older humans and rodents after periods of consumption extending for several weeks. We investigated the feasibility of utilizing Drosophila as a model organism to study the biological effects of HMB on aging muscle when consumed throughout adult life. Using flight ability as an index of flight muscle function, we found that HMB attenuates the age-dependent decline in flight ability. Male and female flies fed a diet supplemented with 10 mg/mL HMB had significantly higher flight scores from median age until the onset of flight senescence than control flies fed a standard diet. HMB supplementation also resulted in improved flight scores in males before median age and delayed the onset of flight senescence in females. Notably, the consumption of HMB throughout adult life increased the rate of survival and extended lifespan. The effect on lifespan did not result from changes in food consumption or body weight. Old flies on the HMB-supplemented diet retained a higher proportion of flight muscle mitochondria whose morphology resembled that of young flies than the control diet group. Together, these results suggest that HMB attenuates the age-dependent decline in flight ability and prolongs lifespan by enhancing muscle health.

## 1. Introduction

The aging process is characterized by a progressive and simultaneous decline in many bodily functions. A progressive reduction in muscle function with age has been observed in organisms ranging from fruit flies to humans [1]. The decline in muscle function is largely attributed to sarcopenia, a loss of muscle mass [2], and dynapenia, a loss of muscle strength independent of muscle mass [3]. The muscular deficits associated with aging in humans invariably decrease quality of life, hindering the ability to perform the usual activities of daily living, increasing risk of fall and injury, and increasing health-care costs and mortality [4]. Thus, specifically curtailing the rate of decline in muscle function may help mitigate age-related physical and socio-economic consequences. Muscle function and growth is dependent on the availability of amino acids at sufficient cellular concentrations to promote muscle proteostasis; that is, a balance between protein synthesis and degradation [5,6]. Intracellular amino acids are maintained by dietary intake and cellular recycling. When amino acid levels are low, muscle proteolysis is up-regulated to maintain consistent cellular amino acid levels [7]. Both the synthesis of functional proteins and proteolysis of defective proteins are essential for optimal muscle function. Two lifestyle interventions that may improve muscle function and attenuate the negative physical outcomes of the aging process include exercise and dietary protein and amino acid supplementation. These interventions, separately and in combination, have been shown to enhance muscle protein synthesis in both young and old adults [7,8,9,10]. The dietary maximization of muscle protein synthesis depends on a number of factors including the extent of protein-rich food processing [11], the protein source [10,12,13,14], the extent of protein hydrolysis [15], the dose and timing of administration [16,17], and the age and sex of the subject. Supplements that contain a high level of the branched chain amino acid (BCAA), leucine, among other essential amino acids, mixed with a carbohydrate, administered to young men immediately after resistance exercise, have been shown to be the most effective in promoting muscle protein synthesis [7]. Although results have varied, positive responses have also been seen in elderly subjects, suggesting that supplementation with essential amino acids, in tandem with resistance exercise, may be a means to promote healthy aging in both young and old individuals [7,18,19].

At the molecular level, leucine serves as a direct substrate for muscle protein synthesis as well as an activator of protein synthesis through the multi-protein complex mechanistic Target of Rapamycin Complex 1 (mTORC1) [20,21,22,23]. BCAA transaminase (BCAT) converts leucine to keto-isocaproic acid (KIC) [24], which is then reduced to β-hydroxy-β-methylbutyrate (HMB) by either branched chain alpha-ketoacid dehydrogenase (BCKD) or KIC-dioxygenase (KICD) pathways [25]. Dietary HMB supplementation has been shown to have a positive effect on muscle function in several rodent studies and human trials. In rodents, HMB supplementation decreased myofiber atrophy or markers of muscle proteolysis in models of cancer cachexia [26,27,28], sepsis [29,30], immobilization [31,32], steroid myopathy [33], energy restriction [34], and aging [27]. In young adults participating in clinical studies, 3 g of daily HMB supplementation has been shown to increase gains in strength and muscle mass associated with resistance training and decrease muscle damage associated with strenuous exercise [35,36,37,38,39].

A growing body of evidence suggests that HMB supplementation improves lean body mass composition and muscle function in older subjects [40,41,42,43,44,45], and can mitigate loss of lean mass in elderly subjects during periods of bed rest [44,46]. Several mechanisms of HMB action on muscle have been described, including both an anabolic mechanism through an up-regulation of protein synthesis via the mTOR pathway [26,47], and an anti-catabolic mechanism through a down-regulation of muscle protein breakdown via the ubiquitin–proteasome proteolytic pathway [48,49]. Additional studies are needed to pinpoint the molecular mechanism and physiological effects of HMB administration, particularly with respect to natural aging and sarcopenia.

Here, we use *Drosophila melanogaster* to examine the effect of HMB supplementation on muscle function with age. *D. melanogaster* has proven to be an extremely useful model organism for studies of human diseases and aging due to the high conservation of genetic, regulatory, physiological, and metabolic pathways [50,51,52,53]. As in mammals, *D. melanogaster* muscles deteriorate with age; fruit fly flight ability declines significantly around median age (∼42 days old), and is lost completely by old age (>56 days) [54]. The similarities that exist between *Drosophila* indirect flight muscle (IFM) and mammalian skeletal muscle allow for the examination of conserved mechanisms in aging muscle that could reveal targets for therapeutic and nutritional interventions [54]. We report that a dietary dose of 10 mg/mL HMB, supplied throughout adult life, is sufficient to provoke a physiological response in *D. melanogaster* that attenuates the age-associated decline in flight ability while extending lifespan. These effects are accompanied by an improvement in flight muscle mitochondrial morphology.

## 2. Results

*D. melanogaster* exhibits a progressive loss of flight ability with age [54]. Previously, the supplementation of 10 mg/mL of HMB was observed to significantly attenuate the aging-associated loss of flight in both sexes [55]. However, this effect was observed in flies reared at 20 °C and in flies fed an HMB-supplemented diet beginning at the larval stage [55]. Here, we tested if the effect was also seen in flies reared at 25 °C and fed an HMB-supplemented diet post eclosion.

### 2.1. HMB Supplementation Attenuates Aging-Associated Loss of Flight Ability

HMB supplementation of 10 mg/mL was shown to significantly delay the aging-associated loss of flight at 25 °C. This result was observed in both sexes, as was the case in the previous study [55].

**Figure 1:**
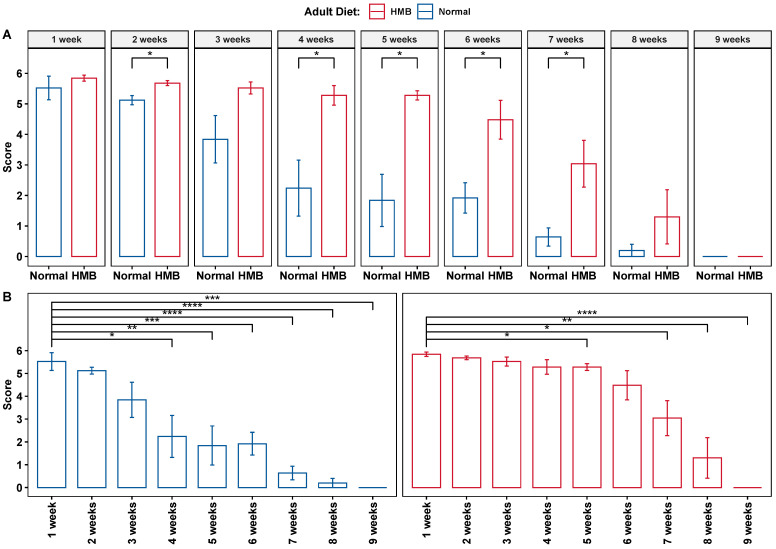
Effect of HMB supplementation on male flight ability. Long-term HMB supplementation attenuates aging-associated loss of flight ability by significantly improving flight ability in median-aged males. (**A**) Flight scores of HMB-supplemented (red) and control diet (blue) flies compared within age groups. All flight scores were evaluated with Student’s *t*-test (95% CI, two-tailed distribution) and error bars represent SE, without any corrections. (**B**): Flight scores of groups within the same diet, comparing flight scores across age groups. HMB-supplemented males (**right panel**) show delayed onset (at 5 weeks) of the significant decline in flight ability when compared to their normal-diet-reared counterparts, who showed a significant decline at week 4 onwards (**left panel**). * = (*p* < 0.05), ** = (*p* < 0.01),*** = (*p* <0.001), **** = (*p* < 0.0001).

Compared to flies raised on a normal diet, flies raised on an HMB (10 mg/mL)-supplemented diet after eclosion had significantly higher flight scores at median ages (week 4–7, Figure 1A). Males raised on a normal diet showed a significant and sustained decline in flight scores beginning at week 4 when compared to their younger (1 week) counterparts. HMB supplementation delayed this effect by 1 week (Figure 1B). This was also reflected in the median age (2 and 4–6 weeks) flight scores of HMB-supplemented flies, which were higher than those of flies raised on regular diet (Figure 1). Flies raised on a 10 mg/mL HMB diet post eclosion had a significantly higher flight scores at week 2 (5.68 ± 0.098 vs. 5.12 ± 0.388 *p*-value < 0.05), week 4 (5.28 ± 0.32 vs. 2.24 ± 0.917 *p*-value < 0.05) week 5 (5.28 ± 0.17 vs. 1.84 ± 0.854 *p*-value < 0.01), and week 6 (4.48 ± 0.64 vs. 1.92 ± 0.496 *p*-value < 0.01) (Table A1).

**Figure 2:**
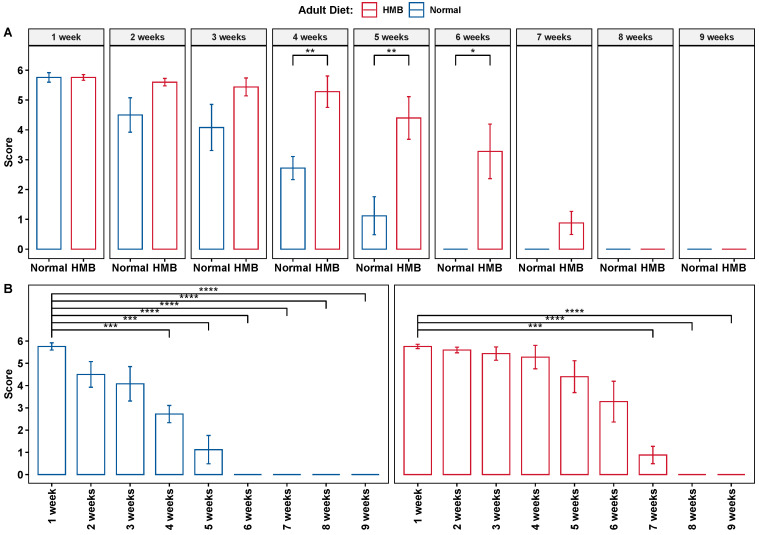
Effect of HMB supplementation on female flight ability: Long-term HMB supplementation in adults attenuates aging-associated loss of flight ability by significantly improving flight ability in median-aged females. (**A**) Flight scores of HMB-supplemented (red) and control diet (blue) female flies, compared within age groups. All flight scores were evaluated with Student’s *t*-test (95% confidence interval (CI), with two-tailed distribution) and error bars represent SE, without any corrections. (**B**): Flight scores of groups within the same diet, comparing flight scores across age groups. HMB-supplemented females show a delayed onset of significant decline in flight ability when compared to their normal-diet-reared counterparts, who showed a significant decline from week 4 onwards. * = (*p* < 0.05), ** = (*p* < 0.01),*** = (*p* < 0.001), **** = (*p* < 0.0001).

Similar trends were observed in female flies. Females flies raised on an HMB-supplemented (10 mg/mL) diet also showed significantly higher flight scores at week 4 (5.28 ± 0.528 vs. 2.72 ± 0.388, *p*-value < 0.01, Student’s *t*-test with two-tailed distribution), week 5 (4.4 ± 0.716 vs. 1.12 ± 0.637, *p*-value < 0.01, Student’s *t*-test with two tailed distribution) and week 6 (3.28 ± 0.916 vs. 0, *p*-value < 0.01, Student’s *t*-test with two-tailed distribution) (Figure 2 and Table A2). Supplementation with HMB also preserved the flight ability of females for longer as a function of age. This was observed when comparing the onset of the significant decline in flight scores. In the control group, the first significant reduction in flight score occurred at week 4. In HMB-supplemented flies, the first significant reduction in flight score was delayed by 3 weeks, occurring at week 7. Furthermore, females raised on a normal diet lost their ability to fly by week 6 onward (i.e., flight score of 0), while females raised on an HMB diet maintained their flight ability for 2 more weeks, with the onset of flight senescence occurring at week 8 onward (Figure 2).

### 2.2. HMB Supplementation Extends Lifespan in D. melanogaster

**Figure 3:**
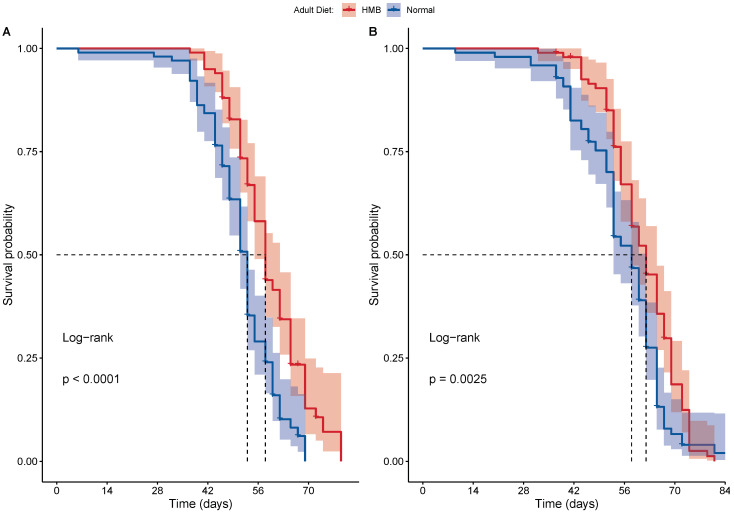
Effect of HMB supplementation on rate of survival: both males and females exhibit a significantly improved lifespan when supplemented with HMB. (**A**): Kaplan–Meier curves of female flies raised on normal and HMB-supplemented diets, shown with 95% Confidence interval (CI). *p*-value calculated with Log rank test. (**B**): Kaplan–Meier curves of male flies raised on normal and HMB-supplemented diets shown with 95% CI. *p*-value calculated with Log rank test. Dashed lines indicate median survival.

Post eclosion, adult lifelong dietary supplementation with HMB (10 mg/mL) extended lifespan *significantly* in *D. melanogaster*. This extension of lifespan was observed in both males and females under standard rearing conditions. A less pronounced lifespan extension effect was also observed in flies reared at 20 °C [55]. The average median lifespan of females raised on an HMB-supplemented diet was 58 days compared to 53 days for those reared on the control diet (Figure 3A). Males similarly showed an improved median survival at 62 days compared to 58 days (Figure 3B). The rate of survival was also significantly different (Figure 3). The Kaplan–Meir curves showed that both males (*p*-value = 0.0025) and females (*p*-value < 0.001) had a significantly improved survival probability when fed HMB (as assessed via log rank test).

### 2.3. HMB Supplementation Does Not Alter Feeding Behavior

Since dietary restriction is a known instigator of lifespan extension, we tested for the possibility that HMB supplementation may alter feeding behavior in flies. We tested this in two ways: with the Capillary Feeding Assay [56] and by measuring wet and dry body weights. The feeding assay was conducted over a period of 48 h following 24 h of acclimatization.

**Figure 4:**
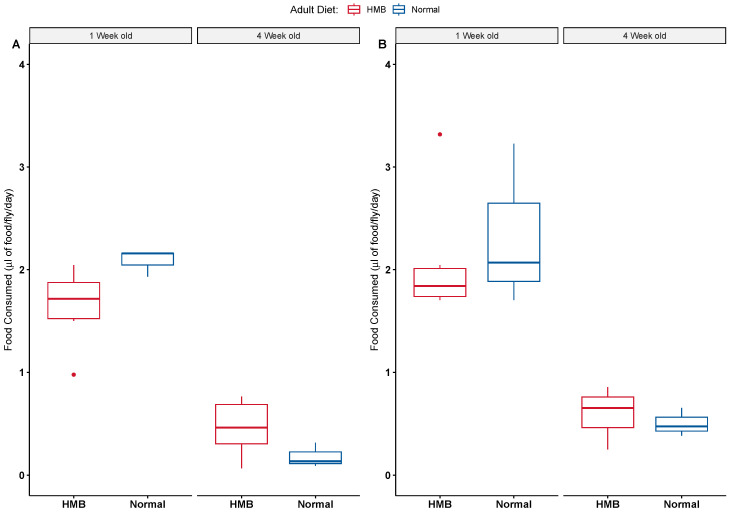
HMB supplementation does not alter food consumption: food consumption showed no significant difference within age groups in both females (**A**) and males (**B**). Food intake was normalized to number of the flies, and corrected for evaporation, n = 5 for each age, diet, and sex group, with three replicates of each. Flies were allowed to acclimate for 24 h before the measurements were taken. Aging led to a significant decline in food intake in both sexes (*p*-value < 0.001, post-hoc tukeyHSD test).

In males, we saw no effect of diet on food consumption rates (Figure 4B), while age showed a significant effect at 24 h (*p* < 0.001, two-way ANOVA). At the 48 h mark, we also observed a significant interaction between age and adult diet (*p* < 0.047). Similar trends were observed in females, wherein adult diet had no effect on food consumption rates (Figure 4A), whereas age showed a significant effect on food consumption (*p* < 0.001, two-way ANOVA). In females, we observed a significant interaction between adult diet and age at both the 24 h (*p* = 0.003) and 48 h marks (*p* = 0.039). To further investigate the effects, we conducted a Tukey HSD.

In males, age leads to a significant decline in food consumption when observed at both the 24 h and 48 h marks (Table A3 and Table A4). In females, we saw similar effects of age on the rate of food intake. The only significant effect of HMB supplementation was observed in females at old age, wherein females showed a significantly higher food intake when consuming HMB-supplemented food (Table A5). This difference as only seen during the 24 h observations and the effect disappears at 48 h (Table A6). Altogether, we saw no effect of HMB supplementation on food consumption in *D. melanogaster* (Table A7, Table A8, Table A9 and Table A10).

**Figure 5:**
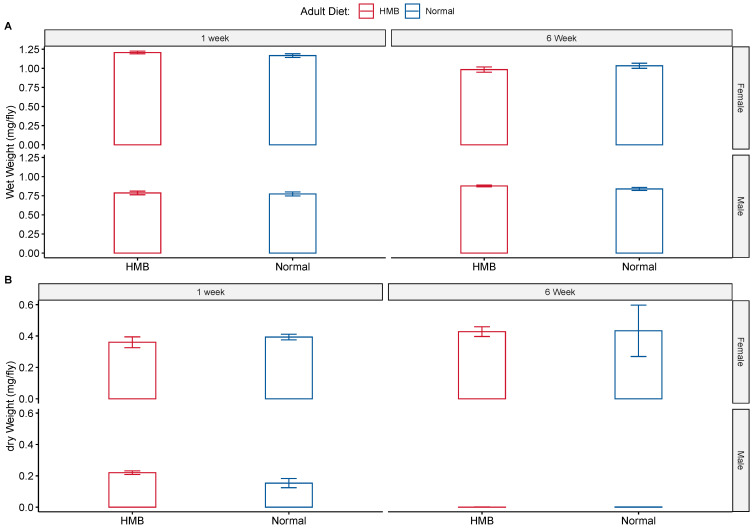
Effect of diet on body weight: HMB supplementation had no effect on adult body weight in either wet (**A**) or dry (**B**) body weight measurements. Three-way Anova indicated that flies show significant differences in body weights according to sex and age, with females being significantly heavier than males (*p* < 0.01, pairwise *t*-test with Bonferroni correction post hoc test) in both dry and wet measurements. Age caused a significant reduction in the wet body weight of females and males (*p*-value < 0.05, pairwise *t*-test with bonferroni correction post-hoc test).

HMB supplementation had no effect on body weight (Figure 5). Young males exhibited an average wet body weight of 0.773 ± 0.042 mg while young males fed with HMB had an average wet body weight of 0.787 ± 0.046 mg. This number increased significantly with age, wherein males on a normal diet showed an average wet weight of 0.839 ± 0.035 mg while HMB-supplemented males tipped the scales at 0.878 ± 0.019 mg. The average dry body weight of young males was 0.22 ± 0.0462 mg, while HMB-supplemented young males tipped the scales at 0.22 ± 0.0416 mg. Average body weight decreased significantly with age, with old males showing an average dry body weight of 0.0019 ± 0.0347 mg and HMB-supplemented males having an average weight of 0.0011 ± 0.0616 mg.

Similar to males, we did not observe an effect of HMB on female body weight. Young females have an average wet body weight of 1.17 ± 0.042 mg and an average dry weight of 0.393 ± 0.0416 mg, while HMB-supplemented flies are not significantly different, showing an average wet body weight of 1.21 ± 0.031 mg and an average dry body weight of 0.36 ± 0.0306 mg. Aging causes a significant decline in both body weight counts. Old female flies on a normal diet weigh 1.03 ± 0.058 mg, (wet) and 0.433 ± 0.0577 mg, (dry), while HMB-supplemented flies have an average weight of 0.983 ± 0.06 mg, (wet) and 0.428 ± 0.0601 mg (dry).

Results consistent with the CAFE assay were observed in body weight measurements, wherein age brought about a decline in body weight (Figure 5). Both dry and wet weights showed significant differences between sexes (*p* < 0.001) when analyzed with ANOVA. Age is also a significant cause for the drop in body weight across both sexes and diets (*p* < 0.05, ANOVA). A significant effect of age is seen on body weight (both wet and dry) in males (Table A11 and Table A12) and females (Table A13 and Table A14), but HMB supplementation shows no effect when analyzed with two-way ANOVA.

### 2.4. HMB Supplementation Confers No Resistance to Oxidative Stress

#### 2.4.1. Resistance to Paraquat Stress

Oxidative stress has been identified as a likely contributor to aging in flies and other organisms [57]. We evaluated whether HMB supplementation provided resistance to applied oxidative stress, which could explain the extended service life and improved flight. To test this, we exposed the flies raised on both diets to paraquat at ages 1 through 4 weeks.

**Figure 6:**
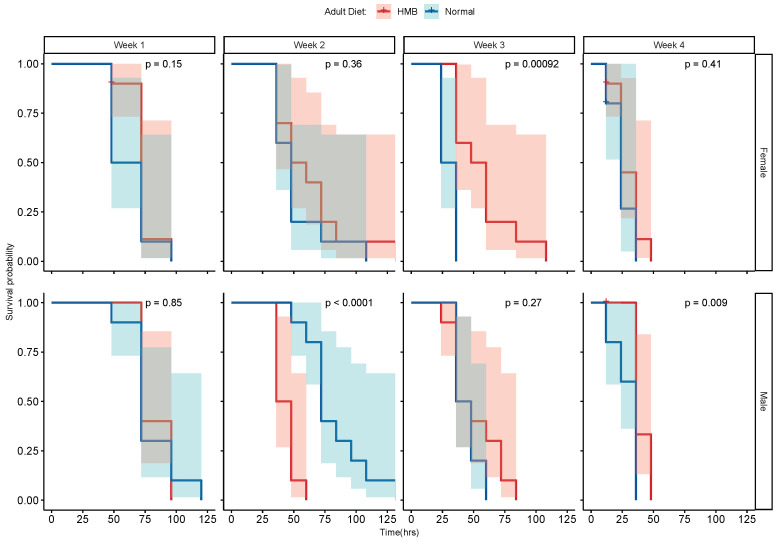
Effect of diet and age on resistance to paraquat: the log rank test on survival curves shows no beneficial effect of HMB supplementation on resistance to paraquat-induced oxidative stress. In males, a significant increase in rate of survival was seen at week 4 in HMB-supplemented flies only, while females raised on an HMB diet showed a significantly higher rate of survival at week 3 only.

Males and females exhibited a similar age-associated decline in resistance to paraquat-induced oxidative stress (Figure 6). HMB supplementation did not show a consistent pattern of providing resistance to paraquat-induced oxidative stress in either sex. However, an age-specific benefit was observed in male flies in week 2 (Figure 6 bottom panel), whereas three-week-old females raised on a normal diet exhibit stronger resistance to oxidative stress (Figure 6 top panel).

#### 2.4.2. Resistance to Hydrogen Peroxide

**Figure 7:**
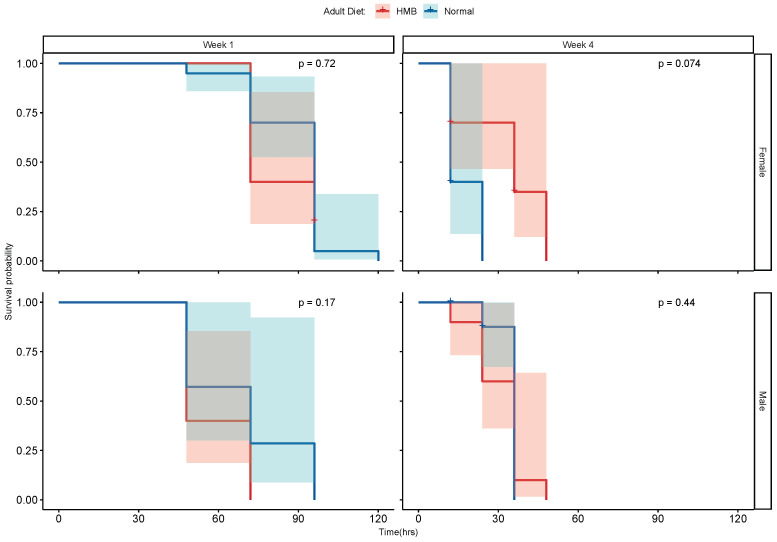
Effect of diet and age on resistance to hydrogen peroxide: the log rank test shows no beneficial effect of HMB supplementation on resistance to hydrogen peroxide-induced oxidative stress in males or females at young and middle age.

To assess whether HMB supplementation increases resistance to oxidative stress triggered by other insults, we repeated the stress experiments with 10% hydrogen peroxide in 1 week and 4 week old flies. In neither males nor females did we observe a clear pattern of differences in the rate of survival (Figure 7). The only differences observed in the rate of survival were due to age.

### 2.5. HMB Supplementation Improves Mitochondrial Morphology in Aging Flies

**Figure 8:**
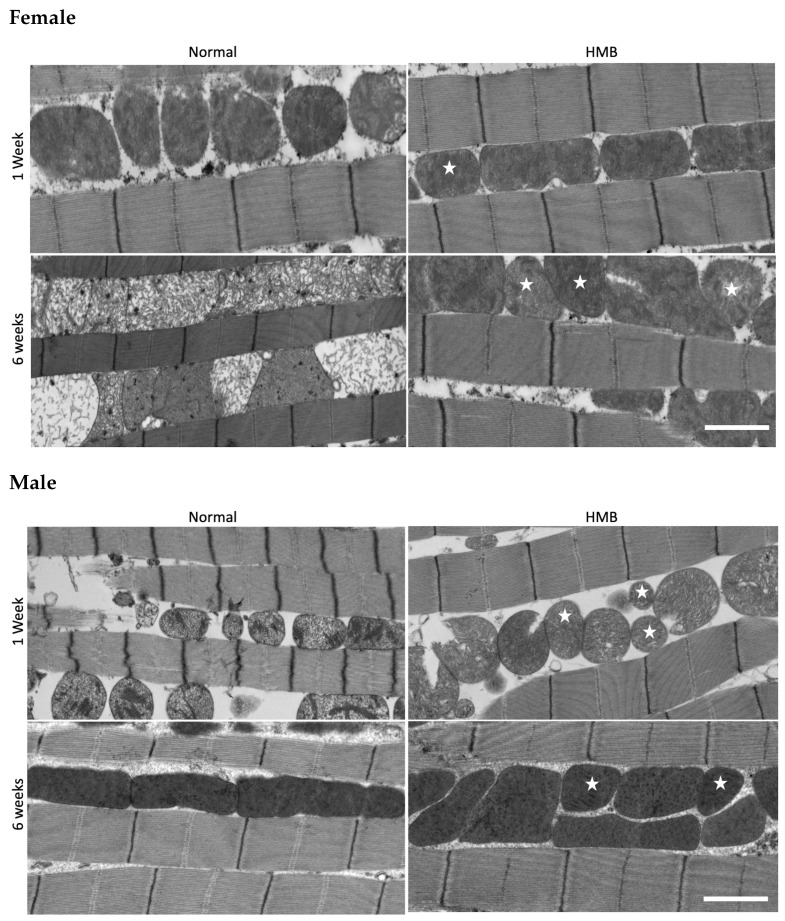
Electron Micrographs of IFM: long-term HMB supplementation improves the prevalence of smaller mitochondria. The top panel shows the EM image IFM of females supplemented with/without HMB at various ages, and bottom panel shows the EM image IFM of males supplemented with/without HMB at various ages. In both sexes, smaller mitochondria (examples of which are indicated with white stars) and an overall higher density of mitochondria were observed. scale bar = 2 μm.

Given the strong effect of HMB supplementation in delaying flight senescence and extending lifespan, we next examined its effect on the properties of the IFM mitochondria. Notable age-associated changes in mitochondrial size and shape were evident in both male and female flies (Figure 8). In particular, larger-size mitochondria appear to be more prevalent in older (6 weeks) flies as compared to younger (1 week) flies. To quantify this effect, we measured mitochondrial area and the ratio of the major to minor axis (see Section 4). Specifically, we observed a significant increase in the prevalence of smaller mitochondria (under 2–3 μm^2^) in male and female flies raised on an HMB-supplemented diet.

**Figure 9:**
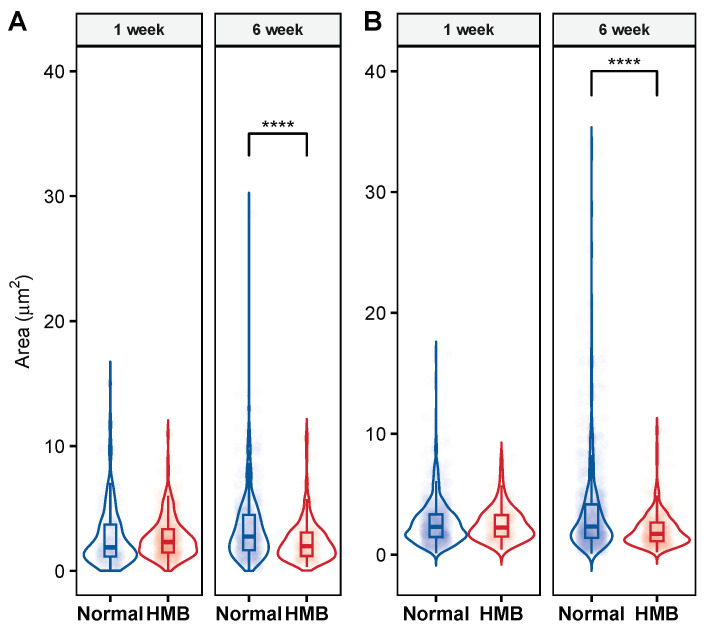
Effect of HMB supplementation on IFM mitochondrial area: long-term HMB supplementation improves the prevalence of smaller mitochondria. (**A**) The distribution of mitochondrial area in female flies fed different diets across the two age groups. Female flies fed an HMB-supplemented diet show a higher prevalence of smaller mitochondria and a significant (**** *p* < 0.005) overall reduction in average mitochondrial area at 6 weeks of age. (**B**): The distribution of mitochondrial area in males flies fed different diets across two age groups. Male flies fed an HMB-supplemented diet show a higher prevalence of smaller mitochondria at six weeks of age and a significant (**** *p* < 0.005) overall reduction in average mitochondrial area with age.

In males, we find that long-term HMB supplementation preserves the overall distribution of IFM mitochondrial area in old age and there is a significant improvement in the prevalence of smaller mitochondria. Although age has a significant effect (*p* < 0.0001, Tukey HSD) on mitochondrial size distribution (Table A16), males fed an HMB- supplemented diet show markedly smaller mitochondria (*p* < 0.0001, Tukey HSD) compared to their normal-diet-raised counterparts (Figure 9B). At 6 weeks, flies supplemented with HMB showed a significantly higher distribution of smaller mitochondria, resulting in a lower average area of 2.06 ± 0.072 μm^2^, while their normal-diet-raised counterparts’ average area was 3.44 ± 0.123 μm^2^. Mitochondrial area in HMB-supplemented flies significantly decreased from 2.51 ± 0.098 μm^2^ at 1 week to 2.06 ± 0.072 μm^2^ at 6 weeks of age (*p* = 0.0657, Tukey HSD), and mitochondrial area from flies fed a normal diet significantly increased from 2.71 μm^2^ at 1 week to 3.44 ± 0.123 μm^2^ at 6 weeks (*p* < 0.001) (details in Table A17).

Similar trends were seen in females, wherein HMB supplementation was observed to attenuate the increased prevalence of larger mitochondria with age. Aging has a significant effect, changing the prevalence of smaller mitochondria (*p* < 0.001); HMB supplementation preserves the prevalence of smaller mitochondria in aged flies (*p* < 0.0001, Tukey HSD) (Figure 9A). At 6 weeks, flies supplemented with HMB showed a significantly higher distribution of smaller mitochondria, with an average area of 2.36 ± 0.121 μm^2^, while the average area of mitochondria from flies fed a normal diet was 3.44 ± 0.124 μm^2^. The mitochondrial area of flies fed the HMB-supplemented diet varied from 2.62 ± 0.072 μm^2^ at 1 week to 2.36 μm^2^ ± 0.121 at 6 weeks of age (*p* < 0.001,Tukey HSD), while the mitochondrial area from flies fed a normal diet varied from 2.71 μm^2^ ± 0.163 at 1 week to 3.44 μm^2^ ± 0.124 at 6 weeks (*p* < 0.0001, Tukey HSD) (details in Table A18).

**Figure 10:**
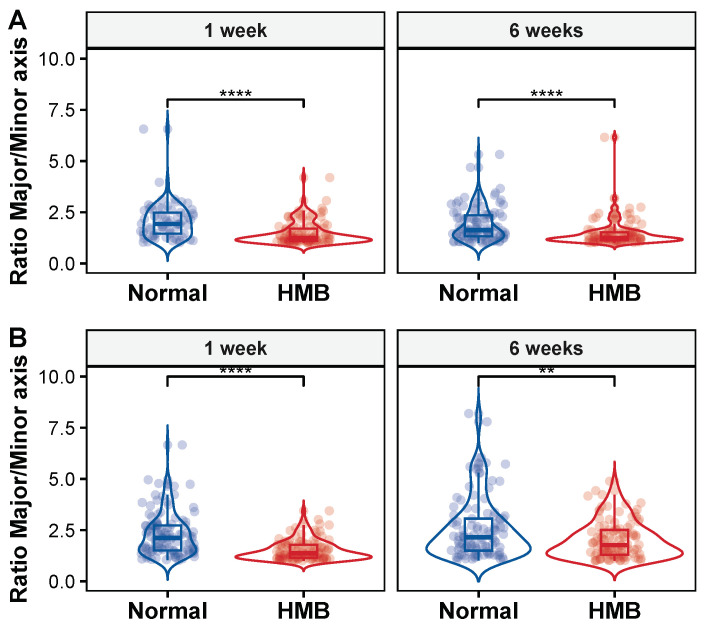
Effect of HMB supplementation on axes ratio: HMB supplementation significantly alters the mitochondrial morphology, which is evident in the differences in axes ratios. (**A**) In females, the ratio in young flies raised on a normal diet is 2.05 ± 0.87 and 1.9 ± 0.69 at six weeks, while in flies supplemented with HMB, the ratio is 1.46 ± 0.59 in young flies and remains relatively constant at 1.45 ± 0.707 at six weeks. The ratio is significantly different (**** *p* < 0.0001, and multiple pairwise *t*-test with BH correction, two tailed distribution). (**B**) In males, HMB supplementation leads to a significantly smaller ratio between the major and minor axis, reflecting a preponderance of rounder mitochondria. The ratio is significantly different (** *p* < 0.01, **** *p* < 0.0001 and, multiple pairwise *t*-test with BH correction, two tailed distribution) in young age, a difference that is maintained in old age. The ratio in young flies raised on a normal diet was 2.27 ± 0.58 and increased to 2.56 ± 1.49 at six weeks, while in HMB-fed flies the ratio was 1.51 ± 0.58 in young flies and increased to 2.02 ± 0.89 at six weeks.

Another measure of mitochondrial morphology that correlates with mitochondrial health is the axis ratio. We found that HMB supplementation has a significant impact on the length ratio of the major/minor axes. Since mitochondria closely resemble an ellipse, we quantified how the ratio of the major axis (longest axis in ellipse) vs. minor axis (shortest axis in ellipses) changed in response to age and HMB supplementation. In females, the ratio decreased from 2.05 at one week to 1.9 at six weeks (Figure 10A) in flies raised on a normal diet. HMB supplementation caused the ratio to be significantly smaller (*p* < 0.01, two-tailed distribution) in young age flies (1.47) and this was maintained through old age (1.45) (Figure 10A). In males, the ratio increased from 2.28 to 2.56 as a function of age in flies fed a normal diet. HMB supplementation reduced the ratio in both young flies (1.51) and in old flies (2.03 (Figure 10B). In both males (Table A19) and females (Table A20), we tested the changes in ratio with a *t*-test and found the differences to be significantly different (*p* < 0.01, multiple pairwise comparison, corrected with BH correction). Overall, we found that mitochondria in HMB-supplemented flies tend to be rounder (i.e., the ratio is closer to 1) at a young age and this is maintained as a function of age.

### 2.6. HMB Supplementation Does Not Improve Citrate Synthase Activity in Aging Flies

**Figure 11:**
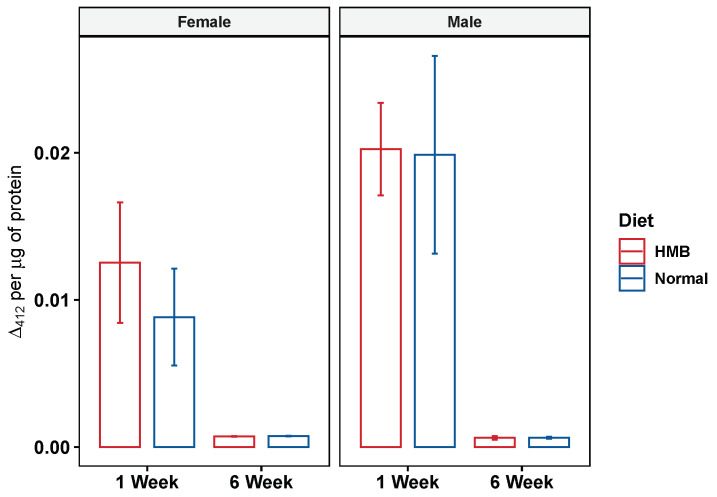
Effect of HMB supplementation on citrate synthase activity: long-term HMB supplementation was not shown to lead to a significant improvement in citrate synthase activity levels in females (**left panel**) or males (**right panel**). All samples were evaluated using two-way ANOVA.

The increased prevalence of smaller mitochondria seen in flies fed HMB could be indicative of a healthier mitochondria population [58,59]. To test for this, we measured the enzymatic activity of citrate synthase. We found that long-term HMB supplementation brings no significant improvement in citrate synthase enzyme activity levels. This is evident in the fact that while age leads to a significant decline in the citrate synthase enzyme activity (analyzed with two-way anova), HMB supplementation does not affect the activity levels in either males or females (Figure 11). Age was a significant factor in explaining the decline in citrate synthase activity levels in both males and females (*p* < 0.001, two–tailed distribution) but diet was not a factor when analyzed by two-way anova.

## 3. Discussion

Aging-associated declines in muscle function reduce quality of life and can shorten lifespan. Numerous studies (reviewed in [60,61]) on dietary interventions and ergogenic supplements, notably HMB, have shown improvements in muscle performance and provided novel insights into the cellular mechanism by which supplements exert their effects. Here, we report that long-term HMB dietary supplementation post eclosion brings substantial benefits during aging *D. melanogaster*. We found that an HMB supplementation of 10 mg/mL significantly attenuated the aging-associated decline in flight ability and significantly improved flight scores in median-age males and females. Additionally, we found that HMB supplementation increases the rate of survival. Median age increased by approximately 9% in females and 7% in males. These improvements did not result from changes in food consumption, body weight, or resistance to oxidative stress. From these results, we conclude that the lifespan extension observed in fruit flies did not result from dietary restriction (i.e., reduced food consumption resulting from aversion to HMB), an intervention shown to extend lifespan in a variety of animal species, including *D. melanogaster* [62]. Furthermore, the data presented here suggest that the extension of lifespan observed in flies raised on an HMB-supplemented diet may be the consequence of improved muscle health, as evidenced by the significant delay in flight senescence and maintenance of mitochondria.

We observed that HMB supplementation brings a substantial change in IFM mitochondrial area and morphology (quantified by axis ratios), in both males and females. HMB effectively preserves the prevalence of smaller IFM mitochondria as a function of age, an effect that likely underlies the delay in flight senescence [54]. Increase in the population of smaller IFM mitochondria has been correlated to increased lifespan in Drosophila overexpressing parkin [58]. In contrast, HMB supplementation had no counteracting effect on the decline in citrate synthase activity observed in aged flies raised on a normal diet. The enzyme activity measurements were done in crude thorax lysates. Previous studies have shown that measurements done on isolated organelles can potentially exacerbate aging effects on muscle [63]. In contrast, studies done with non-aged rats showed an HMB-induced increase in citrate synthase activity in red gastrocnemius fibers but not in white gastrocnemius fibers [64]. The metabolic strategy of IFM is more like that of white skeletal muscle fibers, relying on glycolysis to sustain rapid bursts of activity despite their abundant mitochondria [65]. Thus, it is possible that mitochondrial biogenesis/dynamics in white fast fibers is less responsive to HMB than in red slow fibers. Alternatively, the HMB effect seen on mitochondrial morphology in aging flies is not sufficiently robust to withstand the harshness of the homogenization protocol.

A gradual shift in mitochondrial morphology in muscles as a function of age has been reported in humans, mice, and *D. melanogaster* [1,66,67]. Specifically, in *D. melanogaster*, the preservation of mitochondrial morphology was previously linked to the extension of lifespan [58,59]. Additionally, there is evidence that the genetic stimulation of spargel (srl), the *D. melanogaster* homolog of PGC-1α, considered the master regulator of mitochondrial biogenesis, improves physical activity [68]. Results from studies examining the effect of HMB on mitochondrial content in cultured cells have been mixed, with some studies showing a stimulatory effect, possibly through the upregulation of PGC1-α [69], while others show a reduction in mitochondrial content [70]. HMB also has been shown to stimulate mitochondrial OXPHOS and dynamics when administered to older adults in combination with resistance exercises following 10 days of bed rest [71]. The results presented here, showing a beneficial effect of HMB supplementation on IFM mitochondrial morphology in vivo, should encourage further investigation in other animal systems.

## 4. Materials and Methods

### 4.1. D. melanogaster Rearing and Lifespan Assay

Wild type (Oregon R) flies were raised on standard cornmeal, sugar, and yeast (CSY) medium at 25 °C under standard conditions (12 h light dark cycle, 70% relative humidity, 20 flies per vial, food changed every 2–3 days). For lifespan assay, we followed the recommendations in [72]. Briefly, flies 3–5 days-old were incubated in a egg collection cage with grape agar plate and live yeast paste overnight (16 h). The next day, fresh, non-yeasted grape agar plates were introduced. Flies were allowed to lay eggs on these fresh plates for 24 h. The plates were then washed 4X with PBS (pH 7.1) and 32 μL of eggs were aliquoted with a wide bore pipette tip onto bottles of standard food. The bottles were then placed back in the incubator.

Twenty four hours post eclosion, flies were collected and transferred to either a diet of standard (normal) food or standard food mixed with 10 mg/mL of Ca-HMB (Combi Blocks, QC1944). They were kept in their respective diets for 72 h, after which they were separated by sex under light anesthesia (CO_2_) and transferred to vials containing their respective diets. Each vial had 20 flies. Flies were transferred to fresh vials every 2–3 days, with the dead ones being counted. Live flies stuck in food and flies accidentally killed while being transferred were censored from the study.

The estimate of survival from lifespan data was analyzed using the Kaplan–Meir method [73] and *p*-value was calculated using the log-rank test.

### 4.2. Flight Performance

At weekly intervals, flies were collected under light anesthesia and transferred to individual vials. We collected 5 flies for each dietary group and sex. They were then assigned random numbers and allowed to recover for 24 h. Post recovery, in a dark room, flies were introduced in the center of a transparent acrylic flight chamber with light shining on top and their trajectory was used to assign them a score of 6 (up), 4 (horizontal), 2 (down) or 0 (no flight) [schematic described in Appendix A] [54]. Flight index was determined using the following formula:

6(U/T)+4(H/T)+2(D/T)+0(N/T)

where U, H, D, and N are the total number of flights in each category and T is the total number of flies tested for that group. The flight scores were separated by sex, diet and age, and the data were analyzed using Student’s *t*-test. We compared the flight scores within each age group, stratified by sex. Furthermore, within each dietary group flight scores were compared to the young flies (1 week old), stratified by sex, to observe the effects of age on flight ability. Since the comparisons were pre-decided, we consciously decided to steer away from correcting for multiple comparisons.

### 4.3. Body Weight Measurements

Ten adult flies from each group were anesthetized and weighted on a Sartorius microbalance. These flies were then dried in a drying chamber containing Drierite at 25 °C for 24 h. Post drying, the flies were reweighed as above to obtain the dry body weight. Average body weight was recorded by dividing the total weight by the number of flies. Three trials were conducted for each age group, sex, and diet type.

Since *D. melanogaster* exhibits known sexual dimorphism, we separated the data by sex and measurement type, and analyzed the effects of diet on body weight with a two-way ANOVA. Post hoc comparisons were made with the Tukey HSD (Honest Significant Differences) test.

### 4.4. CAFE Assay

To quantify food intake, we conducted a capillary feeding assay as described in [56]. Briefly, 4 flies were collected in each age group and transferred to a 35 mL vial containing 5% freshly made agar base. Feeding solutions consisted of 5% sucrose and 5% yeast extract, which were boiled briefly before food dye (FD&C 40, 50 μL) and 10 mg/mL HMB were added. Control solutions did not have the HMB. Capillary tubes were filled with 5 μL of these solutions and inserted on top of the vials, allowing for the flies to access the food upside down. Each vial had two capillary tubes.

Flies were introduced in this setup and left for 24 h to acclimatize. After this, fresh feeding tubes were inserted and the drop in capillary was taken every 12–24 h. An evaporation control consisted of a vial with a similar setup but without the flies. Total food consumed was calculated as

$$\frac{\sum \Delta (ExperimentalCapillaries)-\sum \Delta (EvaporationControl)}{\sum FliesPerTube}$$

The food consumption data were gathered over a period of 48 h. The data were stratified by sex and time point and analyzed with a two-way ANOVA, with post hoc comparisons being made with the Tukey HSD test.

### 4.5. Stress-Resistance Assay

Ten flies from each age group were sorted under light anesthesia (CO_2_) and allowed to recover for 24 h in separate vials containing either control diet (standard CSY medium) or control diet supplemented with 10 mg/mL HMB, corresponding to their prior diet. After that, flies were transferred to empty vials containing filter paper disks soaked in 300 μL of either 5% sucrose or 5% sucrose plus 20 mM Paraquat (cat. no. 856177, Sigma–Aldrich, St. Louis, MO, USA). Dead and alive flies were counted every 12 h and flies that became stuck were censored.

A similar procedure was followed for H_2_O_2_ assay except, instead of paraquat, we used 10% H_2_O_2_ (*w*/*v*). The rate of survival was visualized using a Kaplan–Meir survival curve.

### 4.6. Electron Microscopy

Adult flies were dissected under CO_2_ and fixed mostly as described [74], except where noted. Briefly, adult fly thoraces were fixed in Karnovsky’s buffer followed by sequential dehydration with ethanol and fixation with propylene oxide. The sections were visually inspected under light microscope, and sections that had visibly healthy IFM fibers were further processed by embedding and contrasted with uranyl acetate. These sections were then laid on a copper grid and imaged with the JOEL electron microscope at 80 kv.

### 4.7. Image Analysis

The imaged micrographs of adult fly thoraces were analyzed with Fiji^TM^, ver 1.53t image analysis software (obtained from https://imagej.net/software/fiji/ accessed on 23 September 2022). The micrographs were surveyed and, within a section, mitochondria whose entire perimeter was fully visible were selected. We chose to include only mitochondria that were fully visible and clearly distinguishable in a longitudinal section. Using a free-hand drawing tool from the Fiji software, the outline of the mitochondria was marked. Total mitochondrial area was determined for each mitochondria using the area tool in Fiji^TM^. Similarly, two bisecting straight lines were drawn connecting the widest and longest distance of mitochondria to generate the axes’ length. The longest axis was designated the major axis and the shortest the minor axis. The ratio of major/minor axis length was also calculated. Sections from at least 5 flies per treatment, separated by age and sex, were evaluated.

### 4.8. Citrate Synthase Assay

Adult flies were dissected under CO_2_ and thoraces crushed with a chilled dounce homogenizer (type A) in ice-cold isolation buffer (250 mM sucrose, 10 mM Tris-HCl pH 7.4 and 0.15 mM MgCl_2_). The crude lysate was centrifuged at 500× *g* for 1 min at 4 °C. The supernatant was transferred into fresh chilled tubes and centrifuged at 10,000× *g* for 5 min at 4 °C. The pellet was re-suspended in 100 μL isolation buffer.

The assay was conducted using a Citrate Synthase assay kit (Sigma, Kanagawa, Japan, Cat no: CS0720) as per manufacturer’s instructions in a 96 well plate, using a plate reader (Tecan Ltd., Dorset, UK) in kinetic mode at 10 s intervals. The total citrate synthase activity per well was determined from the the linear rate of change in absorbance over 90 s.

The total citrate synthase activity was normalized to total protein content per sample, which was determined using a BCA Assay (Thermo Fisher, Waltham, MA, USA, Cat no: 23225). All the lysates generated during the dissection step were analyzed in 3 technical replicates and each group was assayed in 3 independent replicates (n = 60 for each group). Twenty flies were dissected per age, treatment, and sex group.

### 4.9. Statistical Analysis

All plots were generated and an analysis was conducted using R programming language. Survival curves were analyzed and generated using the Survminer, ver 0.4.8 accessed on 20 December 2023 [75] and Survival, ver 3.8.3, accessed on 12 December 2024 Package [76,77] in R. ANOVA and *t*-test were performed with the help of R-statix, ver 0.7.2 accessed on 12 December 2024 package [78] in R.

Plots were generated using the ggplot2 [79] and ggpubr package [80] in R.

## 5. Conclusions

In conclusion, we report that HMB supplementation in Drosophila attenuates the decline in muscle function with age, consistent with studies in humans and other vertebrates. These findings encourage the use of *D. melanogaster* as an appropriate model organism to explore the effects of HMB on age-related muscle deterioration and to uncover the biological mechanisms of such effects. The extent to which the findings of this study are unique to Drosophila is unknown and further validation in other organisms (including model organisms such as *C. elegans* and mice) will be required. We also report the novel finding that HMB supplementation enhances lifespan in *D. melanogaster*. This provides another strategy through which to study the genetic basis of aging in flies and the possible implications for human health.

## Figures and Tables

**Figure A1:**
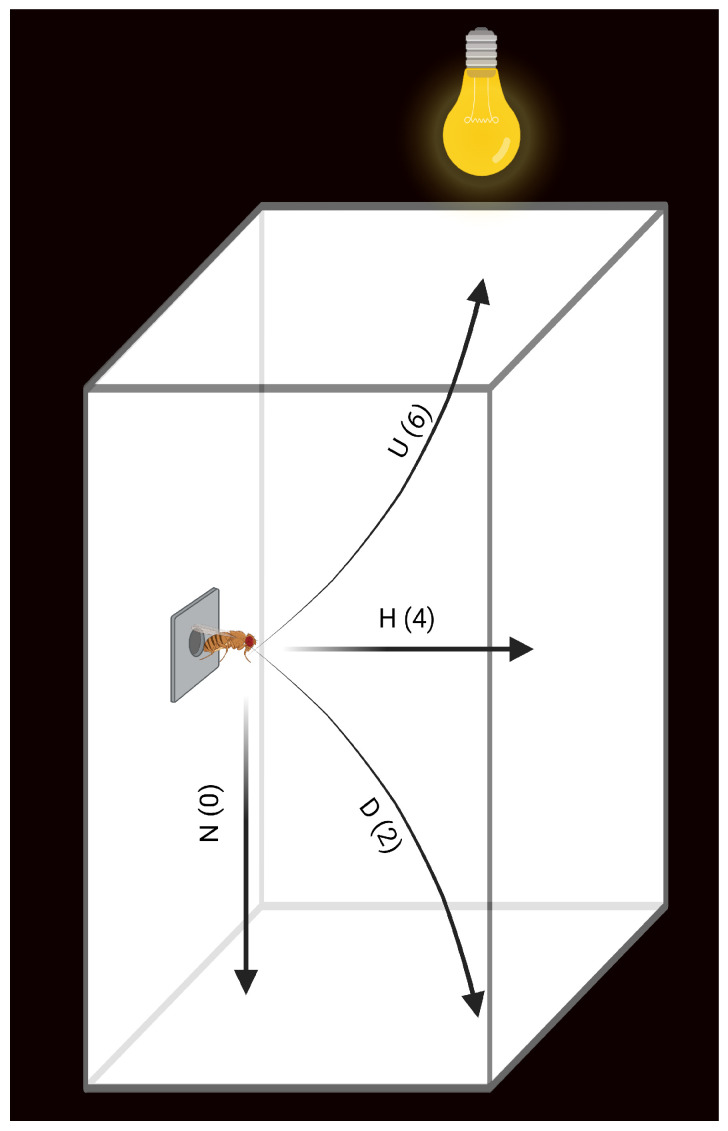
Schematic of flight testing and scoring is depicted in this image. Briefly, a fly is introduced into the chamber which is 30 cm × 30 cm × 40 cm, and kept in a dark room. On top there is a light source. Depending on the flight path of the fly, score is assigned. Image created using BioRender. https://BioRender.com/d89t238 accessed on 12 January 2025.

## Data Availability

The original contributions presented in this study are included in the article. Further inquiries can be directed to the corresponding author.

## Abbreviations

The following abbreviations are used in this manuscript:

BCAA	branched-chain amino acid
CI	confidence interval
CSY medium	cornmeal, sugar, and yeast medium
HMB	β-hydroxy- β-methylbutyrate
IFM	indirect flight muscle
TOR	target of rapamycin

## Appendix A

**Table A1:** Summary Statistics of Flight Scores of Males at 25 °C.

Diet	Flight Score (±SE)	Age
Normal	5.52 (±0.388)	1 week
HMB	5.84 (±0.098)	1 week
Normal	5.12 (±0.15)	2 week
HMB	5.68 (±0.08)	2 week
Normal	3.84 (±0.776)	3 week
HMB	5.52 (±0.196)	3 week
Normal	2.24 (±0.917)	4 week
HMB	5.28 (±0.32)	4 week
Normal	1.84 (±0.854)	5 week
HMB	5.28 (±0.15)	5 week
Normal	1.92 (±0.496)	6 week
HMB	4.48 (±0.637)	6 week
Normal	0.64 (±0.299)	7 week
HMB	3.04 (±0.765)	7 week
Normal	0.2 (±0.2)	8 week
HMB	1.3 (±0.889)	8 week
Normal	0 (±0)	9 week
HMB	0 (±0)	9 week

**Table A2:** Summary Statistics of Flight Scores of Females at 25 °C.

Diet	Flight Score (±SE)	Age
Normal	5.76 (±0.16)	1 Week
HMB	5.76 (±0.098)	1 Week
Normal	4.5 (±0.576)	2 Week
HMB	5.6 (±0.126)	2 Week
Normal	4.08 (±0.774)	3 Week
HMB	5.44 (±0.299)	3 Week
Normal	2.72 (±0.388)	4 Week
HMB	5.28 (±0.528)	4 Week
Normal	1.12 (±0.637)	5 Week
HMB	4.4 (±0.716)	5 Week
Normal	0 (±0)	6 Week
HMB	3.28 (±0.916)	6 Week
Normal	0 (±0)	7 Week
HMB	0.88 (±0.388)	7 Week
Normal	0 (±0)	8 Week
HMB	0 (±0)	8 Week
Normal	0 (±0)	9 Week
HMB	0 (±0)	9 Week

**Table A3:** Tukey HSD summary of 24 h rate of feeding ANOVA in males.

Term	Contrast	Adj *p* Value
Adult Diet	Normal-HMB	7.59 × 10^−1^
Age	4 Week old-1 Week old	8.89 × 10^−6^
Adult Diet:Age	Normal:1 Week old-HMB:1 Week old	8.88 × 10^−1^
Adult Diet:Age	HMB:4 Week old-HMB:1 Week old	7.82 × 10^−4^
Adult Diet:Age	Normal:4 Week old-HMB:1 Week old	2.66 × 10^−3^
Adult Diet:Age	HMB:4 Week old-Normal:1 Week old	1.17 × 10^−3^
Adult Diet:Age	Normal:4 Week old-Normal:1 Week old	2.54 × 10^−3^
Adult Diet:Age	Normal:4 Week old-HMB:4 Week old	9.92 × 10^−1^

We see significant effects of Age negatively influencing the rate of food consumption, while HMB supplementation shows no significant effect.

**Table A4:** Tukey HSD summary of 48 h rate of feeding ANOVA in males.

Term	Contrast	Adj *p* Value
Adult Diet:Age	Normal:1 Week old-HMB:1 Week old	9.448580 × 10^−1^
Adult Diet:Age	HMB:4 Week old-HMB:1 Week old	3.093000 × 10^−4^
Adult Diet:Age	Normal:4 Week old-HMB:1 Week old	2.264880 × 10^−2^
Adult Diet:Age	HMB:4 Week old-Normal:1 Week old	5.646400 × 10^−3^
Adult Diet:Age	Normal:4 Week old-Normal:1 Week old	1.209837 × 10^−1^
Adult Diet:Age	Normal:4 Week old-HMB:4 Week old	5.878281 × 10^−1^

We see significant effects of Age negatively influencing the rate of food consumption, while HMB supplementation shows no significant effect.

**Table A5:** Tukey HSD summary of 24 h rate of feeding ANOVA in females.

Term	Contrast	Adj *p* Value
Adult Diet	Normal-HMB	5.82 × 10^−1^
Age	4 Week old-1 Week old	5.25 × 10^−8^
Adult Diet:Age	Normal:1 Week old-HMB:1 Week old	1.77 × 10^−1^
Adult Diet:Age	HMB:4 Week old-HMB:1 Week old	2.84 × 10^−5^
Adult Diet:Age	Normal:4 Week old-HMB:1 Week old	2.51 × 10^−5^
Adult Diet:Age	HMB:4 Week old-Normal:1 Week old	7.6 × 10^−6^
Adult Diet:Age	Normal:4 Week old-Normal:1 Week old	6.3 × 10^−6^
Adult Diet:Age	Normal:4 Week old-HMB:4 Week old	5.29 × 10^−1^

We see significant effects of Age negatively influencing the rate of food consumption. HMB supplementation shows a significant decline in consumption at old age.

**Table A6:** Tukey HSD summary of 48 h rate of feeding ANOVA in females.

Term	Contrast	Adj *p* Value
Adult Diet	Normal-HMB	2.14 × 10^−1^
Age	4 Week old-1 Week old	4.00 × 10^−3^
Adult Diet:Age	Normal:1 Week old-HMB:1 Week old	9.63 × 10^−1^
Adult Diet:Age	HMB:4 Week old-HMB:1 Week old	3.30 × 10^−2^
Adult Diet:Age	Normal:4 Week old-HMB:1 Week old	6.40 × 10^−1^
Adult Diet:Age	HMB:4 Week old-Normal:1 Week old	3.90 × 10^−2^
Adult Diet:Age	Normal:4 Week old-Normal:1 Week old	4.93 × 10^−1^
Adult Diet:Age	Normal:4 Week old-HMB:4 Week old	5.40 × 10^−1^

We see significant effects of Age negatively influencing the rate of food consumption, while HMB supplementation shows no significant effect.

**Table A7:** ANOVA Summary of 24 h feeding data of Males.

Term	DF	SUMSQ	MEANSQ	Statistic	*p* Value
Adult Diet	1	0.02	0.02	0.10	7.59 × 10^−1^
Age	1	11.47	11.47	45.95	8.89 × 10^−6^
Adult Diet:Age	1	0.12	0.12	0.49	4.94 × 10^−1^
Residuals	14	3.49	0.25	NA	NA

**Table A8:** ANOVA Summary of 48 h feeding data of Males.

Term	DF	SUMSQ	MEANSQ	Statistic	*p* Value
Adult Diet	1	0.09	0.09	0.27	6.13 × 10^−1^
Age	1	12.43	12.43	36.06	3.23 × 10^−5^
Adult Diet:Age	1	0.58	0.58	1.68	2.16 × 10^−1^
Residuals	14	4.82	0.34	NA	NA

**Table A9:** ANOVA Summary of 24 h feeding data of Females.

Term	DF	SUMSQ	MEANSQ	Statistic	*p* Value
Adult Diet	1	0.03	0.03	0.32	5.82 × 10^−1^
Age	1	9.08	9.08	109.69	5.25 × 10^−8^
Adult Diet:Age	1	0.52	0.52	6.34	2.46 × 10^−2^
Residuals	14	1.16	0.08	NA	NA

**Table A10:** ANOVA Summary of 48 h feeding data of Females.

Term	DF	SUMSQ	MEANSQ	Statistic	*p* Value
Adult Diet	1	0.22	0.22	1.69	0.21418
Age	1	1.49	1.49	11.52	0.00436
Adult Diet:Age	1	0.05	0.05	0.39	0.53997
Residuals	14	1.82	0.13	NA	NA

**Table A11:** ANOVA Summary of total wet body weight of Males.

Term	DF	SUMSQ	MEANSQ	Statistic	*p* Value
Age	1	0.02	0.02	14.57	0.00411
Adult Diet	1	0.00	0.00	1.62	0.23514
Residuals	9	0.01	0.00	NA	NA

**Table A12:** ANOVA Summary of total dry body weight of Males.

Term	DF	SUMSQ	MEANSQ	Statistic	*p* Value
Age	1	0.10	0.1	99.74	3.62 × 10^−6^
Adult Diet	1	0.00	0.0	3.13	1.11 × 10^−1^
Residuals	9	0.01	0.0	NA	NA

**Table A13:** ANOVA Summary of total wet body weight of Females.

Term	DF	SUMSQ	MEANSQ	Statistic	*p* Value
Age	1	0.10	0.1	33.94	0.000251
Adult Diet	1	0.00	0.0	0.03	0.873853
Residuals	9	0.03	0.0	NA	NA

**Table A14:** ANOVA Summary of total dry body weight of Females.

Term	DF	SUMSQ	MEANSQ	Statistic	*p* Value
Age	1	0.01	0.01	0.44	0.522
Adult Diet	1	0.00	0.00	0.06	0.816
Residuals	9	0.18	0.02	NA	NA

**Table A15:** TUKEY HSD comparison of mitochondrial areas in female.

Term	Contrast	Null.Value	Estimate	Conf.Low	Conf.High	Adj *p* Value
Diet	Normal-HMB	0	0.66	0.43	0.89	2.07 × 10^−8^
Age	6 week-1 week	0	0.21	−0.02	0.44	0.06905
Diet:Age	Normal:1 week-HMB:1 week	0	0.09	−0.37	0.54	0.961
Diet:Age	HMB:6 week-HMB:1 week	0	−0.26	−0.74	0.22	0.501
Diet:Age	Normal:6 week-HMB:1 week	0	0.82	0.46	1.18	3.1 × 10^−8^
Diet:Age	HMB:6 week-Normal:1 week	0	−0.35	−0.91	0.21	0.38
Diet:Age	Normal:6 week-Normal:1 week	0	0.73	0.27	1.19	2.49 × 10^−4^
Diet:Age	Normal:6 week-HMB:6 week	0	1.08	0.60	1.56	6.5 × 10^−8^

**Table A16:** TUKEY HSD comparison of mitochondrial areas in male.

Term	Contrast	Null.Value	Estimate	Conf.Low	Conf.High	Adj *p* Value
Diet	Normal-HMB	0	0.89	0.64	1.14	7.19 × 10^−11^
Age	6 week-1 week	0	0.46	0.23	0.69	8.91 × 10^−5^
Diet:Age	Normal:1 week-HMB:1 week	0	0.15	−0.40	0.70	0.9033
Diet:Age	HMB:6 week-HMB:1 week	0	−0.45	−1.04	0.15	0.2089
Diet:Age	Normal:6 week-HMB:1 week	0	0.93	0.40	1.46	4.657 × 10^−5^
Diet:Age	HMB:6 week-Normal:1 week	0	−0.60	−1.03	-0.16	2.675 × 10^−3^
Diet:Age	Normal:6 week-Normal:1 week	0	0.78	0.43	1.14	6.9026 × 10^−8^
Diet:Age	Normal:6 week-HMB:6 week	0	1.38	0.96	1.80	6.43 × 10^−11^

**Table A17:** Summary stats for male mitochondrial area.

Age	Diet	Variable	n	Mean	Median	S.E
1 week	HMB	Area	201.00	2.51	2.25	0.10
1 week	Normal	Area	661.00	2.66	2.30	0.07
6 week	HMB	Area	384.00	2.06	1.72	0.07
6 week	Normal	Area	874.00	3.44	2.34	0.12

**Table A18:** Summary stats for female mitochondrial area.

Age	Diet	Variable	n	Mean	Median	S.E
1 week	HMB	Area	497.00	2.62	2.31	0.07
1 week	Normal	Area	215.00	2.71	1.89	0.16
6 week	HMB	Area	186.00	2.36	1.98	0.12
6 week	Normal	Area	471.00	3.44	2.75	0.12

**Table A19:** Pairwise comparison results of *D. melanogaster* male mitochondrial major and minor axes.

Age	Axes	Group1	Group2	n1	n2	Statistic	df	*p*	*p*.adj
1 week	Major	HMB	Normal	111	69	−7.30	110.55	0.00	0.00
6 weeks	Major	HMB	Normal	102	104	−6.77	147.41	0.00	0.00
1 week	Minor	HMB	Normal	111	69	−0.33	132.19	0.74	0.74
6 weeks	Minor	HMB	Normal	102	104	−2.80	153.80	0.01	0.01

**Table A20:** Pairwise comparison results of *D. melanogaster* female mitochondrial major and minor axes.

Age	Axes	Group1	Group2	n1	n2	Statistic	df	*p*	*p*.adj
1 week	Major	HMB	Normal	111	69	−7.30	110.55	0.00	0.00
6 weeks	Major	HMB	Normal	102	104	−6.77	147.41	0.00	0.00
1 week	Minor	HMB	Normal	111	69	−0.33	132.19	0.74	0.74
6 weeks	Minor	HMB	Normal	102	104	−2.80	153.80	0.01	0.01
